# DGKH-PA-mTOR: A pharmacologically druggable metabolic axis underpinning tyrosine-kinase-inhibitor resistance in hepatocellular carcinoma^☆^

**DOI:** 10.1016/j.iliver.2025.100198

**Published:** 2025-11-01

**Authors:** Feifan Cai, Bixing Zhao

**Affiliations:** a The United Innovation of Mengchao Hepatobiliary Technology Key Laboratory of Fujian Province, Mengchao Hepatobiliary Hospital of Fujian Medical University, Fuzhou 350025, Fujian, China; b College of Biological Science and Engineering, Fuzhou University, Fuzhou 350108, Fujian, China

We reviewed the recent work by Loh et al., which identified diacylglycerol kinase eta (DGKH) as a pivotal driver of cancer stemness/self-renewal and resistance to tyrosine-kinase inhibitors (TKIs) in hepatocellular carcinoma (HCC).^1^ In addition to establishing DGKH as a pivotal driver of HCC stemness and TKIs resistance, they delineated a DGKH-phosphatidic acid (PA)-mammalian target of rapamycin (mTOR) signaling axis and demonstrated that E1A-associated protein p300 (EP300) transcriptionally up-regulates DGKH expression. Collectively, the study identify DGKH as a central regulatory node in HCC progression and nominate it as a promising therapeutic target (Fig. 1).

**Fig. 1:**
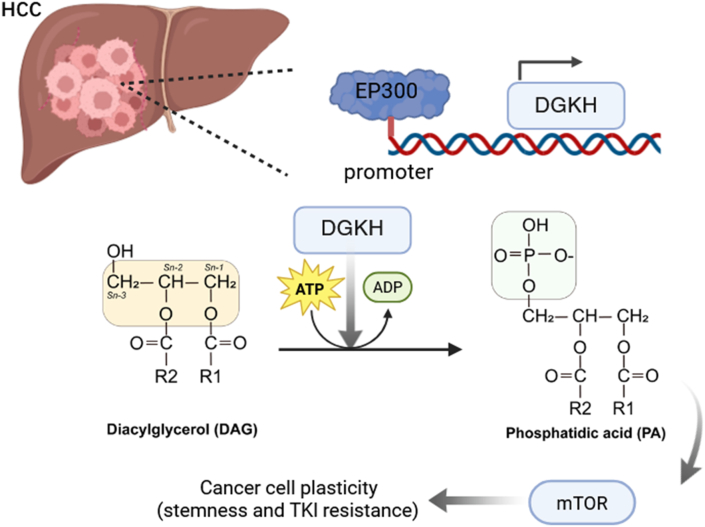
Proposed model illustrating how DGKH-driven phosphatidic-acid oncometabolism sustains cancer stem-cell traits and tyrosine-kinase inhibitor resistance in hepatocellular carcinoma. Created by BioRender. Cai,F.(2025) https://app.biorender.com/illustrations/68ceae5633db9d7f9546630d. HCC, hepatocellular carcinoma; EP300, E1A-associated protein p300; DGKH, diacylglycerol kinase eta; ATP, adenosine triphosphate; ADP, adenosine diphosphate; TKI, tyrosine kinase inhibitor; mTOR, mammalian target of rapamycin.

This study is grounded in a well-characterized spectrum of resistance mechanisms to TKIs in HCC, especially against sorafenib and lenvatinib. Previous work has mapped a complex network of escape routes that encompass (i) gain-of-function oncogenic mutations, (ii) compensatory activation of bypass signaling cascades, (iii) epigenetic reprogramming, and (iv) adaptive remodeling of the tumor microenvironment.^2^, ^3^, ^4^, ^5^ Although reprogramming of lipid metabolism is increasingly recognized as a driver of HCC initiation, progression and chemoresistance, its specific contribution to TKIs resistance remains understudied. Loh et al. close this knowledge gap by embedding lipid metabolism squarely within the established TKI-resistance framework. They show that DGKH dependent production of PA ignites mTOR activity independently of the canonical phosphatidylinositol 3-kinase-protein kinase B (PI3K-AKT) axis, thereby forging a direct mechanistic link between lipid signaling, cancer stem-cell traits and sustained mTOR activation.

While long recognized as a central intermediate in membrane phospholipid metabolism, evidence now positions PA as a critical second messenger. It has been shown to bind a conserved sequence within the mTOR FK506 binding protein 12 (FKBP12)-rapamycin binding domain, acting as a physical scaffold that facilitates substrate recruitment and kinase activation.^6^ Crucially, Loh et al. provides causal evidence that PA is sufficient to independently drive stemness and drug resistance, suggesting the potential development of PA probes or PA competitive inhibitors as more specific intervention strategies compared to mTOR inhibitors.^1^

Besides, metabolic intervention can convert TKI regimens from cytostasis to cytotoxicity. DGKH depletion shifts sorafenib's effect from growth arrest to robust induction of cell death; in immunocompetent mice, combining DGKH depletion with sorafenib reduces tumour burden and depletes residual cancer stem-cell compartments.^1^ This approach aligns with the broader concept of metabolic sensitization. For example, exogenous β-hydroxybutyrate (β-HB) reprograms tumour metabolism reducing lactate and shifting from aerobic glycolysis to ketone utilisation—thereby reversing sorafenib resistance and synergising with regorafenib.^7^ Likewise, the metabolic enzyme aldo-keto reductase family 1 member B1 (AKR1B1) promotes multidrug resistance through glucose–lipid rewiring and antioxidant defences; pharmacologic inhibition with the aldose-reductase drug epalrestat mitigates lenvatinib resistance.^8^ Together with Loh et al., these studies consolidate a paradigm in which targeting metabolic circuits enhances tumour susceptibility to existing therapies.

Despite the notable advances positioning the DGKH-PA-mTOR axis as an emerging mechanism of resistance, several important questions warrant further investigation.

1. Clinical validation in large, multicentre cohorts should test associations between DGKH/PA and outcomes (objective response rate, progression-free survival) under TKI therapy and evaluate prognostic utility.
2. Non-invasive biomarkers warrant development: sensitive lipidomics to quantify circulating PA or extracellular vesicles phosphatidic acid (EV-PA), and extracellular vesicles carrying DGKH or phosphorylated mTOR effectors as liquid-biopsy readouts to track resistance dynamics.
3. On the drug development front, highly selective DGKH inhibitors are not yet available. Current DGK inhibitors are broad and only partially active across isoforms.^9^ However, the recent entry of a first-in-human, highly selective diacylglycerol kinaseζinhibitor (BAY 2965501) into clinical trials is a pivotal development. This breakthrough does more than demonstrate feasibility; it provides a powerful pharmacological precedent and a potential structural blueprint for the rational design of DGKH-targeted agents,^10^ encouraging structure-guided design against the DGKH catalytic pocket.
4. Combination strategies-pairing PA-biogenesis inhibitors (beyond DGKH, e.g., acylglycerol kinase) with rapalogs or ATP-competitive mTOR inhibitors alongside first-line TKIs-should be optimised for dosing, schedule, and therapeutic window to maximise synergy and minimise toxicity.

In conclusion, Loh and colleagues place the lipid-metabolizing enzyme DGKH at the center of therapeutic resistance in HCC and propose clinically actionable combination strategies. The DGKH-PA-mTOR axis, as a metabolism-stemness pathway, represents a tractable avenue for precision combination therapy in HCC. As targeted agents, immunotherapies, and interventional approaches advance in parallel, precise interventions directed at the metabolism-stemness axis may offer a tractable route to overcoming tyrosine-kinase inhibitor resistance.

## CRediT authorship contribution statement

**Feifan Cai:** Writing – original draft, Conceptualization. **Bixing Zhao:** Writing – review & editing, Writing – original draft, Conceptualization.

## Data availability statement

Not applicable.

## Declaration of generative AI and AI-assisted technologies in the writing process

During the preparation of this work the authors did not use generative AI or AI-assisted technologies.

## Funding

The authors received no financial support to produce this manuscript.

## Declaration of competing interest

All authors have declared no conflict of interest.
